# Supplementary material for: MetaRibo-Seq measures translation in microbiomes
Source: Nat Commun. 2020 Jun 29;11:3268. doi: 10.1038/s41467-020-17081-z (PMC7324362; doi:10.1038/s41467-020-17081-z)
Supplement: Supplementary file 10 — Supplementary Data 7 [file 41467_2020_17081_MOESM10_ESM.zip › File2/Confidence_High_Taxonomy/324550_out.krona.html]

Javascript must be enabled to view this page.

members
magnitude
magnitudeUnassigned
count
unassigned
taxon
rank

324550\_out

2

2
superkingdom
2

1224
phylum
2

2
class
28216

order
2
80840

2
family
995019

genus
2
40544

2
species
1262977

SRS019267\_contig\_number\_8669SRS143342\_contig\_number\_14020
